# The osmo-metabolic approach: a novel and tantalizing glucose-sparing strategy in peritoneal dialysis

**DOI:** 10.1007/s40620-020-00804-2

**Published:** 2020-08-07

**Authors:** Mario Bonomini, Victor Zammit, José C. Divino-Filho, Simon J. Davies, Lorenzo Di Liberato, Arduino Arduini, Mark Lambie

**Affiliations:** 1 Department of Medicine, Section of Nephrology and Dialysis, G. d’Annunzio University, Chieti-Pescara, Chieti, Italy; 2 Warwick Medical School, University of Warwick, Clinical Sciences Research Institute, Coventry, England; 3 Division of Renal Medicine, CLINTEC, Karolinska Institutet, Stockholm, Sweden; 4 Faculty of Medicine and Health Sciences, Keele University, Staffordshire, UK; 5 Department of Research and Development, CoreQuest Sagl, Tecnopolo, Bioggio, Switzerland

**Keywords:** Peritoneal dialysis, Glucose-sparing, Solution, Carnitine, Xylitol

## Abstract

Peritoneal dialysis (PD) is a viable but under-prescribed treatment for uremic patients. Concerns about its use include the bio-incompatibility of PD fluids, due to their potential for altering the functional and anatomical integrity of the peritoneal membrane. Many of these effects are thought to be due to the high glucose content of these solutions, with attendant issues of products generated during heat treatment of glucose-containing solutions. Moreover, excessive intraperitoneal absorption of glucose from the dialysate has many potential systemic metabolic effects. This article reviews the efforts to develop alternative PD solutions that obviate some of these side effects, through the replacement of part of their glucose content with other osmolytes which are at least as efficient in removing fluids as glucose, but less impactful on patient metabolism. In particular, we will summarize clinical studies on the use of alternative osmotic ingredients that are commercially available (icodextrin and amino acids) and preclinical studies on alternative solutions under development (taurine, polyglycerol, carnitine and xylitol). In addition to the expected benefit of a glucose-sparing approach, we describe an ‘osmo-metabolic’ approach in formulating novel PD solutions, in which there is the possibility of exploiting the pharmaco-metabolic properties of some of the osmolytes to attenuate the systemic side effects due to glucose. This approach has the potential to ameliorate pre-existing co-morbidities, including insulin resistance and type-2 diabetes, which have a high prevalence in the dialysis population, including in PD patients.

## Introduction

Peritoneal Dialysis (PD) is an established home care, cost-effective kidney replacement therapy, which compared to hemodialysis (HD), offers several advantages including better preservation of residual renal function, a more gradual and continuous solute and fluid clearance, minimal cardiac stress, and similar survival [1]. Several studies over the last decade also show that PD may be a safe and efficient alternative to HD in late-presenting ESRD patients requiring urgent start of dialysis [2]. The dialysate composition includes physiological concentrations of chloride, calcium, sodium, magnesium, and a buffer (lactate and/or bicarbonate). It is also necessary to add an osmotic agent to the solution which will induce water flow, and thereby ultrafiltration, across the peritoneal capillaries via both the water-exclusive aquaporin and the small-pore, solute-coupled fluid pathways [3]. Glucose (molecular weight 180 Da) is the standard osmotic agent used, with concentrations 10-to 50-fold higher than physiological levels [4].

Despite potential benefits, PD is prescribed to only a minority of dialysis patients in certain countries and regions, about 10% in the USA and 13% in Europe [1, 5]. Infectious complications (primarily peritonitis) and catheter problems are the main contributors to reduced technique survival in the short and mid-term. For patients on long term PD, bio-incompatibility of dialysate may represent the main problem. Biocompatibility of a PD solution can be defined as its capacity to leave the anatomical and functional natural characteristics unmodified in time; and it can be divided into local (peritoneum cavity) and systemic. Indeed, it is generally accepted that conventional PD fluids alter the functional and anatomical integrity of the peritoneal membrane over time, by causing inflammation, neoangiogenesis and fibrosis [6]. The progressive damage to the peritoneal membrane is evidenced by faster peritoneal solute transport rate and by a decline of UF capacity eventually resulting in UF failure, which is often characterized by impaired osmotic conductance [7]. More widespread usage of standard dialysate would involve more long-term patients at risk of these problems. Some patients may also develop encapsulating peritoneal sclerosis, a rare but serious complication [8].

Several potential factors have been claimed to be responsible for the poor biocompatibility of PD solutions, including elevated levels of glucose degradation products (GDPs) generated during heat-sterilization of glucose-based solutions, hyperosmolarity, lactate buffer, low pH, and high glucose exposure [9]. In an effort to improve PD, newer, so called biocompatible glucose-based dialysis solutions characterized by neutral or physiological-pH and low-GDP fluids using multi-chamber bags, have been introduced into the market [10] as well as by using other osmotic agents (icodextrin, amino acids) and thereby decreasing the level of glucose exposure. The clinical value of some of the new glucose-based PD solutions, which contain as much glucose as standard solutions, is however still uncertain [11]. PD patients treated with these solutions may experience important benefits such as better preservation of urine volume and kidney function [12], but also a tendency to overhydration likely due to diminished ultrafiltration [13]. In a recent study, Elphick et al. [14] found that use of biocompatible solutions was associated with a slow but progressive rise of peritoneal solute transport rate, which reached levels comparable to those seen with standard dialysate after two years of treatment and thereafter plateaued (while continuing to increase with standard solutions), a potentially beneficial effect that deserves further investigation. Moreover, based on recent findings [15], biocompatibility of neutral -pH, low-GDP fluids cannot be assumed [16]. In children on PD, use of such dialysate was in fact found to induce early peritoneal inflammation, fibroblast activation, epithelial-mesenchymal transition and marked angiogenesis, which determined the PD membrane transport function [15].

Thus, since glucose is the common denominator in the conventional and low-GDP PD solutions, glucose per se, as originally thought, might be the main culprit behind adverse events occurring locally and systemically during the PD lifetime of a patient. Depending mostly on the dialysate volume and glucose concentration used, but also to some extent the peritoneal membrane transport status, PD patients can absorb up to 200 g of glucose per day [17]. Glucose absorption may play a distinct role in the longitudinal changes to the structure and function of the peritoneal membrane observed in some patients [18]. For example, in keeping with the relevance of sodium removal/balance in PD patients, preserving the integrity of the peritoneal membrane function would be key to guarantee an adequate dialytic sodium removal particularly in APD patients [19, 20], and to achieve a better control of volemia and blood pressure [21]. Moreover, excessive intraperitoneal absorption of glucose from the dialysate has many potential systemic metabolic effects, including insulin resistance, new onset diabetes, and cardiovascular disease, which can be attributed to a combination of the effects of carbohydrate and caloric uptake, as well as hyperglycemia [17, 22]. A counter argument could be represented by the obesity paradox, since several epidemiological cohorts have demonstrated that higher body mass index is associated with a reduced mortality risk in patients on chronic hemodialysis [23]. However, no apparent association between obesity and mortality was consistently found for PD patients [24]. One possible reason for this is the presence of glucose in the dialysate, providing regular caloric supplementation that may offset the nutritional advantage of adipose stores to draw on.

Strategies devised to reduce/eliminate glucose-associated toxicity (glucose sparing), represent one of the key objectives of present-day research in PD. In this article, we (i) review the evidence for substances used as alternative osmotic agents to glucose for PD, and (ii) detail the rationale and the results obtained so far for the novel proposed osmo-metabolic approach to the composition of new PD solution [25].

## PD solutions with an alternative osmotic agent

### Commercially available PD solutions

Several alternatives to glucose were examined over the years, but only two agents are currently used in glucose-free dialysate: icodextrin and amino acids. It should be noted that both solutions only replace up to 50% of daily glucose absorption [22].

The colloidal osmotic agent icodextrin is a water-soluble glucose polymer derived from starch, which allows for a slow but sustained peritoneal ultrafiltration and is indicated for use during a single long dwell per day [26]. It is of particular value in high solute transport patients and improves patient’s ultrafiltration without increased risk of adverse effects [27, 28]. Evidence of its benefit on glucose metabolism has been reported in three randomized clinical trials; two in diabetic CAPD [29, 30] and one in non-diabetic APD [31] patients. Both studies with diabetic patients used changes in glycated haemoglobin (HbA1C) as a primary endpoint, and in both studies, a significant reduction in the levels of HbA1C at the end of follow-up was observed. On the other hand, the study of non-diabetic APD patients showed that the use of icodextrin during the long dwell for non-diabetic patients in APD improves insulin resistance by HOMA index, reducing exposure to glucose PD solutions [31].

The clinical benefits of icodextrin appear to stem from better fluid removal as well as of its glucose sparing property. There are several studies suggesting that long-term utilization of icodextrin solution may extend patient and technique survival in PD [32–34]. A very recent systematic review and meta-analysis examined the efficacy and safety of icodextrin versus glucose-only PD regimens [35]. Data analyzed were from 19 randomized controlled trials, enriched with unpublished data from investigator-initiated and industry-sponsored studies. Icodextrin-containing PD solution proved to be associated with clear fluid benefits, such as improvement of peritoneal ultrafiltration and fewer episodes of fluid overload (both with high certainty of evidence). As a matter of fact, icodextrin has been used as a salvage therapy in PD patients with refractory volume overload who were otherwise about to be transferred to HD [36, 37].

The use of icodextrin as a PD fluid may extend time on PD treatment. It is anticipated, therefore, that the share of patients treated with PD will be positively influenced. The relationship between extension of PD treatment time and an increase of the PD treatment share, however, is complex and has been investigated by [38] utilizing the Markov chain model to forecast changes in the development of the ESRD program over time. They concluded that the direct and indirect effects of extended time on PD treatment result in cost savings that could amount to 1.0% of the total cost of dialysis treatment in The Netherlands. Although the relative cost savings are low, the absolute cost savings are substantial. These cost savings would make it possible to offer dialysis treatment to more patients with equal budgets. In The Netherlands, the cost savings would allow for 452 more HD patient years or 635 more PD patient years in the 10-year model period.

However, further high quality, adequately powered randomized clinical trials of sufficient duration are required to accurately establish whether the use of icodextrin solution helps patients to stay on PD longer or live longer [12].

Amino acid based PD solutions (e.g. Nutrineal®) offer the possibility of improving the poor nutritional status of some patients. However, due to the risk of acidosis and azotemia, their use has to be limited to one peritoneal exchange per day [39]. Nutrineal® is a glucose- and GDP-free amino acid-based (0.1%) solution. Accordingly, long-term use (3 years) resulted in normalized protein nitrogen in the bodies of patients. This was accompanied by a decrease in plasma triglycerides [40, 41] and phosphate levels [41–43]. Other studies [44–46] have shown that Nutrineal® is a more biocompatible PD solution than glucose-based ones. Specifically, Asola et al. [47] have shown (using [^11^C]methylaminoisobutyrate -based PET) that muscle amino acid uptake is markedly increased when patients are treated with 1.1% amino acid PD solutions. Delarue et al. [48] further showed that simultaneous carbohydrate intake by patients receiving Nutrineal® PD treatment have a more positive nitrogen balance, indicating that the timing of a once-a-day amino acid based PD infusion relative to the patient’s main meal is important. Canepa et al. [49] used a combined glucose–aminoacid PD solution for 6 months in 8 pediatric APD patients. They found an improvement of anthropometric parameters, particularly those indicating muscle mass and fat stores without modifications of nitrogen waste products were observed. Subsequently Canepa et al. [50] reported a further study using an automatic PD cycler to infuse glucose and AA solutions (proportion of 3:1) simultaneously in pediatric PD patients during the night [50]. This resulted in favorable effects on protein synthesis, hyperinsulinemia, prevention of hyperaminoacidemia, and on maintenance of adequate non-protein calorie/nitrogen ratio (115.4:1) of the absorbed glucose and AA. Therefore, these findings supported the use of this combined glucose-aminoacid solutions, to improve utilization of AA for protein synthesis while controlling BUN levels [50]. However, it would be of interest to ascertain whether the exposure to combined glucose and amino acid solutions is appropriate for PD patients who are already insulin resistant or overtly diabetic, owing to the gluconeogenic potential of the amino acids.

To evaluate whether changes in substrate and insulin levels that occur during PD have effects on muscle protein dynamics by studying muscle protein synthesis, breakdown, and net balance, a crossover study in which adult PD patients served as their own controls was performed by Garibotto et al. [51]. The forearm perfusion studies associated with the kinetics of 3 H-phenylalanine were performed (1) in the basal state and during PD with dialysates that contained dextrose alone in different concentrations, (2) during PD with dialysates containing dextrose alone or dextrose and AA, and (3) in time controls. This study indicated that in PD patients in the fasting state, the moderate hyperinsulinemia that occurs during PD with dextrose alone causes an anti-proteolytic action that is obscured by a parallel decrease in AA availability for protein synthesis. Conversely, the combined use of dextrose and AA results in a cumulative effect, because of the suppression of endogenous muscle protein breakdown (induced by insulin) and the stimulation of muscle protein synthesis (induced by AA availability). When patients on PD are in a fasting state or in a condition with reduced nutrient intake, muscle mass might be maintained better by the combined use of glucose and AA (suppression of protein breakdown and stimulation of muscle protein synthesis) [52].

Recently, Tjiong et al. [42] in a single, open label, random order crossover study, compared the effects of a mixture of AA and glucose versus glucose only containing dialysate, in 2 periods of 7 days each in eight APD patients. They concluded that CCPD with dialysate composed of a mixture of AA and glucose acutely improves protein metabolism, and that this gain made during the night persists to a considerable extent for 24 h. This may be promising for long-term improvement of nutritional status in selected groups of patients.

Potential benefits of glucose-sparing by above formulations have been reported [22]. More recently, two randomized studies (IMPENDIA, EDEN) evaluated whether a low-glucose regimen improves metabolic control in diabetic patients on PD [30]. Patients were randomized 1:1 to dextrose solutions only (control group) or to a low-glucose strategy (combination of icodextrin, amino acids, and dextrose-based solutions in IMPENDIA; the same but for a different dextrose-based solution in EDEN trial). After 6 months, mean glycated hemoglobin (primary end-point) improved in the intervention group (0.5% difference between groups, *p* = 0.006). The study also demonstrated modest but significant improvements in several lipid measures, including triglycerides, VLDL, and apolipoprotein B, in the glucose-sparing group; these improvements could conceivably reduce atherosclerotic risk in the longer term, though this potential benefit requires an outcome study. However, the authors reported in the intervention group a greater number of deaths and serious adverse events, including several related to extracellular fluid volume expansion [30]. A possible explanation, also expressed by the authors, is that in the pursuit of better metabolic values, investigators used lower concentrations of dextrose-based dialysis fluid, when more hypertonic dialysate was indicated to optimize ultrafiltration. If this were the case, the investigators may not have recognized the association between reduced ultrafiltration and these adverse events. It follows, then, that a preference for less hypertonic dialysate could have contributed to the improved metabolic results seen in the intervention group.

The results of these studies, besides confirming improvements in metabolic control of a glucose-sparing prescription as evidenced by reductions in HbA_1c_, serum VLDL cholesterol, serum triglycerides, and apolipoprotein B, also emphasize the importance of efficacious ultrafiltration and the need for close monitoring of fluid status with any glucose-sparing strategy. Furthermore, the wealth of data, information and experience published with glucose-sparing PD solutions (icodextrin, amino acids) already in the market, strongly indicate that the need for new osmotic agents targeting its effect not only on biocompatibility and fluid balance, but also and equally important, on metabolism, is of utmost importance for the future of PD.

### PD solutions under development

Experience over the last three decades has shown that the development of effective and safe osmotic agents to be used in PD solutions undoubtedly proves challenging. For example, small-molecular-weight candidates (like members of sugar alcohol or monosaccharide or disaccharide families), having a metabolism rate slower than their absorption rate, suffered from hyperosmolar syndromes [52]. Thus, mixtures of smaller doses of osmotic agents have been suggested to minimize the potential for adverse metabolic consequences while maintaining ultrafiltration [22].

This concept is supported by a randomized, 3-months trial in nondiabetic CAPD patients, comparing the effects of a PD solution with a mixture of 0.6% amino acids and 1.4% glycerol replacing two 2.27% glucose-based conventional solutions (which were used in the control group) [53]. Glycerol (molecular weight 92 Da) acts as an energy source by entering the glycolytic pathway through the Krebs cycle. Glycerol concentration in the experimental PD solution (1.4%) was lower than that used in previous studies when glycerol was used as the sole osmotic agent, at a high concentration as needed to result in an adequate fluid balance however putting the patient at risk of a hyperosmolar syndrome [54]. On the other hand, the reduced concentration of AA (0.6%) may allow a twice daily use of the solution, with AA exchange comparable to a single 1.1% amino acid daily exchange [22]. Clinical use of the 0.6% AA/1.4% glycerol PD solution proved to be safe and well tolerated, with a significant reduction of glucose absorption, an ultrafiltration capacity comparable to glucose, and increased dialysate levels of CA 125, the latter possibly indicating better preservation of mesothelial cell mass and, potentially, better biocompatibility [53].

In a subsequent randomized study in a rat model of chronic renal failure [55], daily use for 16 weeks of a bicarbonate/lactate-buffered solution with a mixture of osmotic agents (glycerol 1.4%, AA 0.5%, and dextrose 1.1%) (GLAD) was compared to 3.86% glucose-based solution and also to a buffer solution (bicarbonate/lactate-buffered solution without osmotic agents). Both hypertonic dialysis solution increased peritoneal solute transport, but GLAD exposure was associated with a good preservation of peritoneal morphology in terms of omental vessel density and fibrosis. It is interesting to note that results obtained with the experimental solution were similar to those of bicarbonate/lactate-buffered solution without osmotic agents [55].

Another compound which has been tested for a potential use in the PD solution is Taurine. Taurine (molecular weight 125 Da) is a sulfonic beta-amino acid with high water stability that regulates osmotic balance and ion transport in mammalian cells where it is present in high concentration. In a rat PD model, Nishimura and colleagues [56] examined peritoneal transport and biocompatibility of a new PD solution containing taurine instead of glucose as osmotic agent. Net ultrafiltration of the PD-taurine solution rose in a dose-dependent manner, and the 3.5% concentration was equivalent to the 3.86% standard glucose solution. With respect to the peritoneal membrane, the PD-taurine solution was more biocompatible (lower mesothelial and fibroblast-like cell proliferation) than PD-glucose [56]. However, much caution must be taken if taurine is going to be used as an osmotic agent in PD. Patients with chronic kidney disease (CKD) are reported to be taurine depleted, with low plasma and intracellular concentrations of taurine [57]. Suliman et al. [58] have reported on an open non-randomized trial in 10 chronic hemodialysis patients on the effect of daily oral taurine substitution for 10 weeks on various neurophysiological parameters as well as plasma and muscle levels of taurine. The dose of taurine, 100 mg/kg/day, was comparable to that previously used in human clinical trials. Neurological symptoms (dizziness, non-rotatory vertigo) appeared in 2 out of the first 4 patients. Although these symptoms rapidly disappeared after stopping taurine, long-term risks of excessive accumulation cannot be ruled out either. The taurine levels in these two patients increased far above the normal plasma (5513% and 1780%) and muscle intracellular (239% and 351%) ranges.

Another osmotic agent under development is hyperbranched polyglycerol, a polyether polymer synthesized by polymerization of glycidol that is highly water-soluble, hydrophilic, and chemically stable in aqueous solution [59].

Studies in a rat model of acute (4 h) or chronic (3 months) PD have shown hyperbranched polyglycerol-containing PD fluid, as compared to glucose-based PD solutions, may induce less peritoneal injury and inflammation while achieving similar ultrafiltration and waste removal [60, 61]. A more recent study [62] compared hyperbranched polyglycerol fluid to low-GDP and icodextrin PD fluid in a rat model (obese type 2 diabetic ZSF1 rats) of metabolic syndrome. At the end of the 3 months treatment period, the authors found that PD fluid containing hyperbranched polyglycerol better preserved the structures and function of the peritoneal membrane and kidneys, and induced less systemic adverse effects on metabolism, immune response and serum antioxidant capacity [62]. However, caution must be taken when evaluating studies with icodextrin performed in rats. Garcia-López et al. [63] studied the metabolism of icodextrin and alpha-amylase activity following daily exposure to dialysis solutions containing either glucose or icodextrin as osmotic agent in rats. No icodextrin could be detected in plasma, suggesting that it was metabolized and excreted by the kidney in these non-uremic rats. In contrast to uremic peritoneal dialysis patients, chronic exposure to icodextrin did not seem to further affect alpha-amylase activity or icodextrin metabolism. They concluded that the much higher alpha-amylase activity in plasma and dialysate in rats than in humans explains the much more rapid metabolism of icodextrin in rats compared with PD patients [63]. Results with this new PD solution are promising, but metabolism of polyglycerol in plasma and ramifications of plasma accumulation and tissue disposition with long-term use remain to be precisely determined [4].

A recent study examined theoretically and experimentally in rabbits whether low-polydispersity glucose polymers could have advantages over icodextrin for long-dwell exchange in PD [64]. Icodextrin is a mixture of glucose polymers with a polydispersity (ratio of weight-average to number-average molecular weight) of approximately 2.6, and previous studies had indicated that monodisperse polymers with molecular weight between 2 and 20 kDa might achieve a higher ultrafiltration than icodextrin [65]. Findings of the study show that compared to a 7.5% icodextrin solution, a PD solution containing 11% glucose polymers with a molecular weight of 18–19 kDa and a polydispersity of 2.0 can generate a higher ultrafiltration without any increase in carbohydrate absorption [64].

## The “Osmo-Metabolic” approach to PD solutions

Patients suffering from ESRD may benefit locally and systemically from a glucose-sparing approach, in terms of the PD solutions prescription [22]. Glucose-sparing strategies so far described are based on the use of glucose-free PD fluid, in order to reduce patient’s glucose exposure.

A novel and tantalizing tool to antagonize glucose-associated toxicity is represented by use of osmo-metabolic agents in the PD fluid. We define as osmo-metabolites those substances which exhibit both osmotically and metabolically favorable properties [25, 66]. This approach would ensure glucose-sparing not only by reducing intraperitoneal glucose exposure without compromising ultrafiltration, but also by the independent mitigation of underlying systemic negative metabolic effects caused by the glucose load, a sort of bioactive glucose sparing. Osmo-metabolic agents can be used alone, or in combination to maximize their therapeutic effects. Two such candidate agents are represented by l-carnitine and xylitol. In the following section, we review the available evidence for the use of l-carnitine and/or xylitol in PD fluid as a new glucose-sparing strategy. In order to appreciate the metabolic contribution that these molecules make to the overall condition of the PD patient, it is necessary to describe some basic features of the biochemical mechanisms that underlie their metabolic function.

## **l**-Carnitine

### Biological features

**Fig. 1:**
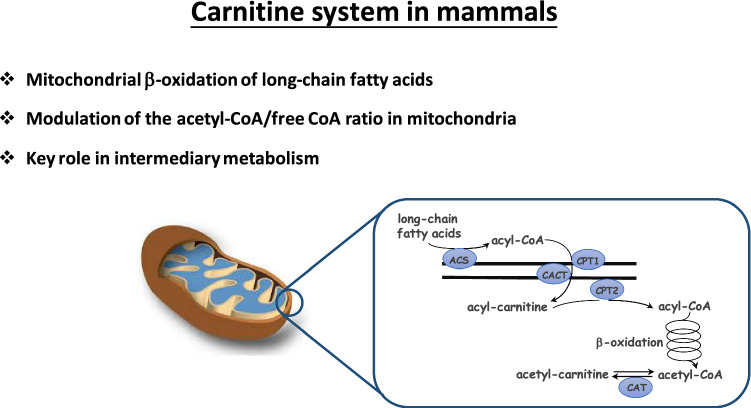
Main metabolic function of the carnitine system. *ACS* acyl-CoA synthetase, *CPT1* carnitine palmitoyltransferase 1, *CPT2* carnitine palmitoyltransferase 2, *CACT* carnitine-acylcarnitine translocase, *CAT* carnitine acetyltransferase

l-Carnitine (molecular weight 161.2 Da) is a naturally occurring compound known to be essential for fatty acid oxidation in the mitochondria [67] (Fig. 1). It is highly water soluble and chemically stable in aqueous solutions in both acidic and basic conditions. l-Carnitine binds acyl groups via ester bonds with carboxylic acids at its 3-hydroxyl position, thus serving as a ‘’carrier” for fatty acid moieties.

l-carnitine and acylcarnitine content is high set in muscle and liver, with a total content in the human body of about 300 mg/Kg, of which more than 95% is intracellular. l-carnitine can be synthesised from the essential amino acid lysine and methionine, though the average adult diet provides about 75% of daily carnitine requirements [67]. In healthy adults, the plasma concentration of total l-carnitine is in the range of 25–50 μM while the plasma level of acetyl-carnitine is 3–6 μM. Approximately, 98–99% of the circulating l-carnitine filtered by the glomerulus undergoes tubular reabsorption with a threshold concentration of about 40–60 μM [68]. Thus, in subjects with normal renal function exogenous administration of l-carnitine raises its renal clearance, to maintain a plasma concentration of about 50 μM [68].

l-carnitine is taken up by cells mostly due to the organic cation transporter-2 (OCTN2) present into the plasma membrane [69]. Loss of function mutation of OCTN2 causes a remarkable reduction of the uptake of L-carnitine into the cells and decreased intracellular l-carnitine concentration in the affected tissues, including liver, skeletal muscle and heart, a rare and severe pathological condition known as primary carnitine deficiency (PCD) [69].

A key partner of l-carnitine is a family of acyltransferases able to catalyse the reversible transfer of acyl groups between coenzyme A and l-carnitine [70]. In mammals, different l-carnitine acyltransferases show typical subcellular location and substrate preference for specific chain-length acyl-CoA esters.

### Metabolic features of l-carnitine relevant to PD therapy

As discussed above, one of the main limitations of PD therapy is the poor global (local + systemic) biocompatibility of glucose, the most common osmotic agent used nowadays. Thus, reducing glucose exposure would not only spare the peritoneal membrane of its potential deleterious effects, but it would also attenuate the adverse systemic actions of glucose. In this regard, the use of l-carnitine as an osmo-metabolic agent/additive may also be envisioned taking into account its metabolic role exerted through a mode of action as a conditional vitamin and/or conditional drug.

l-Carnitine as a conditional vitamin The concept of l-carnitine being a conditional vitamin stems from to the fact that under certain disease states its requirement may exceed dietary intake and/or endogenous synthesis. Except for the rare PCD, some controversy and misconceptions exist regarding the presence of a similar phenotype in secondary carnitine deficiency (SCD). SCD can be caused by increased losses due to a variety of different conditions, including patients in HD and peritoneal dialysis [70, 71].

Heterozygous carriers of the OCTN2 loss of function mutation show a 50% reduction of carnitine transport activity in fibroblasts and show a moderate reduction of plasma carnitine content comparable to subjects affected by SCD [72]. Although heterozygous carriers are thought to be asymptomatic, ageing and additive cardiac risk factors may precipitate cardiac dysfunction, left ventricular hypertrophy and arrhythmias. Indeed, experimental studies in heterozygous rodents have shown that the incidence of cardiomyopathy increases with ageing, the risk for cardiac abnormalities and with reduced survival when additional cardiac burdens are imposed [73, 74].

A genetic epidemiological study in Japan showed that the prevalence of heterozygotes for *SLC22A5* mutations in OCTN2 was 1.01%, and echocardiography revealed that they were susceptible to late onset benign cardiac hypertrophy (odds ratio 15.1, 95% CI 1.39–1.64) compared with wild types [75]. In addition, heterozygosity for PCD mutations has been linked to cardiac arrhythmias that resolve after carnitine treatment [76].

Similar findings were obtained in murine models of moderate carnitine deficiency induced with carnitine depleting agents such as pivalic acid, mildronate and d-carnitine [77–79]. We have shown that rats exposed to a moderate reduction in cardiac l-carnitine content are more susceptible to myocardial injury induced by β-adrenergic stimulation (isoproterenol) along with an unanticipated high mortality rate [77]. When carnitine deficiency was corrected by supplementing the pivalate-treated rats with l-carnitine, the observed detrimental effects of isoproterenol were negligible along with no mortality. A similar outcome was observed in pressure overload-induced cardiomyopathy in heterozygous carrier mice of OCTN2 mutation with a moderate l-carnitine deficiency [74]. Indeed, l-carnitine supplementation of such l-carnitine deficient mice subjected to ascending aortic constriction not only prevented cardiac injury, but it also increased survival rate compared to untreated mice. A moderate heart carnitine deficiency induced by d-carnitine treatment increased sensitivity to bupivacaine-induced asystole, whereas l-carnitine repletion completely reversed such effect [78]. This experimental study was conducted by the same authors after they experienced a case of peri-operative cardiac arrest in a carnitine deficient child (valproate-induced) in the context of intravascular administration of bupivacaine, an anaesthetic known to exert cardiotoxic side effects [78]. Moreover, Roussel et al. were able to reproduce electrophysiological and structural abnormalities observed in patients affected by PCD by inducing a 50% reduction of l-carnitine plasma levels in mice exposed to MET88, a competitor inhibitor of OCTN2 [79]. The authors, therefore, suggest that in the presence of short QT syndrome or unexplained cardiac arrhythmias, leading to cardiac arrest even in adult people, carnitine deficiency must be suspected. Altogether, these studies clearly indicate that although a modest reduction of carnitine content in either heterozygous carrier of PCD or SCD may be considered a benign condition not necessarily associated to a pathological phenotype, the superimposition of an additional insult may lead to severe pathological consequences. Therefore, a conditional vitamin action of l-carnitine in PD patients could be envisioned as a prophylactic measure to ameliorate the above consequences.

l-Carnitine as a Conditional Drug. The concept of l-carnitine being a conditional drug, instead, stems from the possibility of overexposing metabolic carnitine target organs to high levels of carnitine. The well-known metabolic roles of carnitine derive from its involvement in fatty acid oxidation in the mitochondria binding acyl groups via ester bonds with carboxylic acids at its 3-hydroxyl position, to serve as a carrier for fatty acids [67]. Therefore, a key partner of l-carnitine is a family of acyltransferases able to catalyse the reversible transfer of acyl groups between coenzyme A and l-carnitine [70]. Because these carnitine acyltransferases catalyse primarily equilibrium reactions, they are important in modulating, and responding to, changes in the concentrations of acyl-CoA esters within the cellular compartments in which they occur. Consequently, it is possible to alter acyl-CoA levels in tissues by overexposing the metabolic carnitine target organs to high levels of carnitine.

Whereas 98–99% of the filtered l-carnitine in the kidney undergoes tubular reabsorption under normal plasma concentrations, at concentrations higher than 40–60 µM renal excretion is highly increased; thus, preventing significant elevations in plasma l-carnitine level after exogenous administration either orally or intravenously [68]. In ESRD patients the exogenous administration of l-carnitine via a suitable systemic route (e.g. peritoneal dialysis) results in remarkable l-carnitine plasma overexposure proportional to the amount administered.

l-Carnitine overexposure is also expected to be present in organs like skeletal muscle and liver at high concentration. An indirect confirmation that this is the case is the remarkable elevation of plasma acetyl-carnitine levels following the administration of free l-carnitine in PD patients [80]. The presence in mitochondria of the equilibrium enzyme CrAT, increases acetyl-carnitine production at the expenses of acetyl-CoA, due to a mass-action effect driven by the increased l-carnitine concentration. Therefore, the possibility to modulate the intra-mitochondrial acetyl-CoA pool by overexposure of tissues to carnitine, may have favourable metabolic consequences toward glucose and lipid homeostasis in insulin resistance and diabetic patients, because acetyl-CoA plays a key role in the regulation of several important cellular functions such as gluconeogenesis, glucose oxidation, protein acetylation, and steroid and fatty acid biosynthesis. In particular, acetyl-CoA acts as an allosteric activator of pyruvate dehydrogenase (PDH) kinase, which phosphorylates and inhibits the alpha subunit of PDH, the enzyme complex that couples glycolysis to glucose oxidation. Therefore, any intervention aimed to keep in a more active state PDH activity (i.e. inhibition of PDH kinase) should, in principle, favour glucose oxidation which in certain conditions (e.g. relative cardiac ischemia) may provide a more efficient use of substrate for energy formation in the heart. Experimentally, an increased muscle glucose uptake/disposal was found in various in vitro and in vivo studies in non-diabetic and diabetic humans and animal models [68, 80–83]. Another key role of the modulation of intra-mitochondrial acetyl-CoA by carnitine in liver regards the activation of pyruvate carboxylation (PC), a pivotal enzyme in gluconeogenesis [84]. Therefore, a reduction of the mitochondrial acetyl-CoA pool size by raised carnitine levels in liver mitochondria will lead to a less active PC, which in turn will significantly reduce gluconeogenetic flux.

Another possible metabolic advantage of overexposure of tissues to carnitine is the promotion of autophagy. Acetyl-CoA plays a prominent role in controlling longevity and autophagy [85]. The involvement of acetyl-CoA in these key processes is based on its ability to acetylate protein lysine residues in various subcellular compartments, including mitochondria. In addition, a recent study has shown that the specific ablation of muscle CrAT increased tissue acetyl-CoA levels and, consequently, the acetylation of many mitochondrial proteins along with an alteration of both glucose and insulin tolerances [86]. Therefore, a pharmacologically mediated excess of carnitine may moderate protein acetylation by shifting the position of the reaction catalysed by CrAT toward acetyl-carnitine.

### Use of l-carnitine in PD fluid

*Biocompatibility profile* Studies in vitro and in vivo (PD rabbit model) showed PD solution containing carnitine to be more biocompatible than standard glucose solutions and those containing icodextrin, in terms of mesothelial and vascular changes [87]. Addition of l-carnitine to glucose-based PD solutions significantly improved the viability of cultured murine fibroblast L929 [88], a standard toxicity test, suggesting a potentially protective role. Furthermore, addition of l-carnitine to cultured human umbilical vein endothelial cells (HUVECs), significantly improved cell viability, did not affect the percentage of early apoptotic cells, and prevented glucose-induced apoptosis [88].

*Osmotic capacity* Studies in animal models (rat, mouse) of PD have shown that equi-osmolar solutions of glucose and l-carnitine result in a comparable amount of net peritoneal ultrafiltration [88]. The capacity of l-carnitine for ultrafiltration across the peritoneum is supported by our results in four stable CAPD patients receiving, over five consecutive days, for the long-dwell night exchanges PD solution containing glucose 1.5% plus l-carnitine 0.25% instead of a 2.5% glucose-based PD solution. Use of the experimental solution was well tolerated and associated with equivalent or possibly slightly higher net ultrafiltration than that achieved with 2.5% glucose PD solutions, despite the lower osmolarity of the carnitine-containing PD solution [88].

The function of the peritoneal membrane has been well described by the three-pore model, according to which the major transport barrier of the membrane is the capillary endothelium, which contains small, large, and ultra-small pores [89]. This predicts that about 60% of the peritoneal ultrafiltration induced by glucose occurs through the small intercellular pores, and 40% via the transcellular aquaporin-1, the molecular counterpart of ultra-small pores [90]. A similar fluid transport can be expected with l-carnitine, though with a little less ultrafiltration via the small pore pathway (since l-carnitine is slightly smaller than glucose) but identical water movement across aquaporins. Indeed, studies in a mouse knockout model for aquaporin-1 demonstrated that approximately 50% of the ultrafiltration generated by either l-carnitine or glucose contained in the PD fluid reflects facilitated water transport through the ultra-small pores [88]. Notably, l-carnitine significantly increased aquaporin-1 protein expression in HUVECs, and significantly reversed the inhibitory effect of glucose on aquaporin-1 protein levels [88].

*Metabolic effects* Short-term clinical studies performed in CAPD patients indicated the possible, safe use of l-carnitine added to the PD solution. In seven CAPD patients, a dose of 2 g of l-carnitine added to the PD fluid and administered during the nocturnal exchange for two months, was well tolerated, restoring plasma levels (normally diminished in PD patients) of l-carnitine, and ameliorating the lipid pattern in six patients [Bazzato G, personal communication, 1981]. In a clinical trial on five CAPD patients who received 20 mg/kg l-carnitine in the first daily PD solution for a period of 14 days, an improvement in nitrogen balance and a good tolerability were found [Kopple JD, personal communication, 1999].

More recently, we examined in a prospective, multicenter, parallel randomised controlled trial the effects of an l-carnitine-containing PD solution [80]. Nondiabetic ESRD patients on CAPD were randomly assigned to receive 4 months of diurnal exchanges with either a standard glucose-based solution or a solution with identical glucose concentrations enriched with l-carnitine (2 g in each daily bag), the nocturnal exchange in both groups being icodextrin. The primary end-point was change in insulin sensitivity, evaluated by performing a euglycemic hyperinsulinemic clamp. Insulin resistance is often associated with ESRD and may cause enhanced morbidity and mortality through an increased occurrence of cardiovascular disease [91].

The presence of l-carnitine in the PD fluid was well tolerated by all patients, and induced a statistically significant increase in insulin sensitivity, as compared to both baseline and to results obtained in the control group. In addition, a significant increase in fasting plasma insulin levels was found in the control, but not the intervention group. In the latter, plasma levels of l-carnitine markedly increased, achieving an apparent steady state after 30 days suggesting apparent equilibrium between l-carnitine absorption, exposure, and excretion (drained PD fluid and urine). Supra-physiological plasma levels of l-carnitine (> 1 mmol/L), as discussed above, might be behind the insulin-sensitizing effect of the l-carnitine-containing PD solution. At the end of the study period, stability in peritoneal membrane transport parameters and dialysis adequacy were observed. An interesting observation is the significant decrease in daily urine output in the control group, whereas urine volume did not change in the intervention group. Since l-carnitine urinary excretion was increased in l-carnitine-treated patients, this finding might be explained by an osmotically driven maintenance of diuresis by l-carnitine and may have clinical relevance since urine output in PD patients is important in maintaining fluid balance [92].

## Xylitol

### Biological features

Xylitol is a five-carbon sugar alcohol (pentitol, molecular weight 151.2 Da) which is synthesised by the reduction of d-xylulose. Based upon the daily urine excretion of l-xylulose in patients with essential pentosuria [93], approximately 5–15 g of xylitol are formed per day in the human body. Xylitol administered either orally or intravenously is mainly removed by the liver, which is responsible for 50–80% of xylitol metabolism in normal conditions. Extra-hepatic metabolism in the kidney, lung, erythrocyte, fat stores and myocardium can account for about 15–20% of the remaining proportion of parenterally administered xylitol [93, 94]. The clearance of xylitol from blood appeared to be a first-order kinetic process with a half-life of 19–23 min for non-adapted human adults, whereas its excretion is by simple glomerular filtration in the absence of any re-adsorptive mechanism [93].

**Fig. 2:**
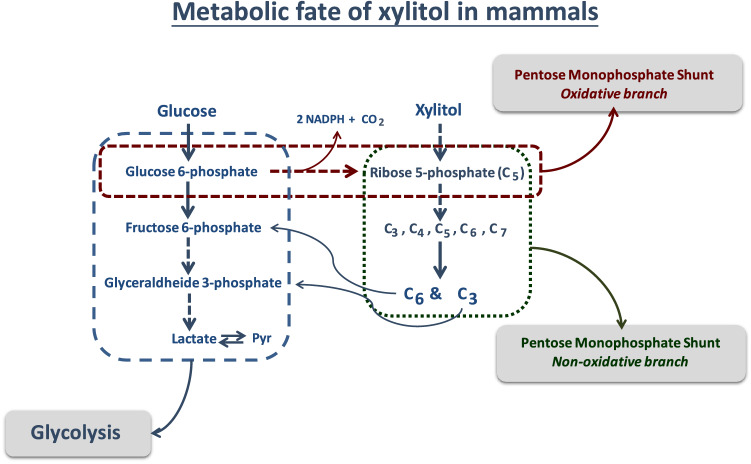
Metabolism of d-xylitol. Thank to the presence of a NAD-dependent xylitol dehydrogenase, d-xylitol is first oxidized to d-xylulose and then phosphorylated to D-xylulose-5-phosphate by a d-xylulose-kinase. The latter phosphorylated product represents the entrance point to the non-oxidative branch of the pentose phosphate shunt, the main metabolic fate of d-xylitol

On entry into the cell, xylitol is first oxidized by the NAD-xylitol dehydrogenase to d-xylulose, causing the NADH/NAD ratio to increase [93, 94] (Fig. 2). Subsequently, the d-xylulose-kinase phosphorylates d-xylulose to d-xylulose-5-phosphate. The latter is an intermediate of the pentose monophosphate shunt and it is metabolized to fructose-6-phosphate and glyceraldehyde phosphate. Consequently, most of the xylitol is rapidly converted to glucose and glycogen, and only small quantities are converted to lactate (Fig. 2).

### Metabolic features of Xylitol relevant to PD therapy

Xylitol is an approved compound in Germany as a sugar substitute of glucose in parenteral nutrition (i.e., Aminomix 4, Fresenius Kabi; Nutriflex Combi, BBraun) and the Ministry of Health authorized a maximal dose of xylitol of 3 g/kg/day. Indeed, xylitol entry into cells is insulin-independent and it is efficiently metabolized after being intravenously infused with no elevation of glycemia, stimulating much less insulin secretion than does glucose [95]. Since 1970, it has been used in diabetics and for parenteral nutrition, as a glucose substitute in critically ill patients (i.e., post-traumatic, septic patients), though in most cases xylitol has been administered in combination with other sugars such as glucose and fructose [93]. An improved glucose homeostasis was observed with hypocaloric xylitol and the mixture of glucose/xylitol (1:1) in animal as well as in human studies [96]. The nitrogen sparing properties of xylitol were observed in surgical intensive care patients. Hepatic gluconeogenesis was significantly reduced when compared to isocaloric glucose during xylitol infusion after trauma and in septic patients. In a more recent case control study, intensive care patients treated for 24 h with a parenteral nutrition product containing only xylitol received significantly less insulin than patients treated with parenteral nutrition products not containing xylitol [97].

In a rat animal model of non-esterified fatty acid (NEFA)*-*induced insulin resistance, Kishore et al. examined the effect of parenteral xylitol infusion during a euglycemic hyperinsulinemic clamp [98]. Elevated NEFA contribute to insulin resistance in humans through such mechanisms as downregulation of insulin-signaling, resulting in impaired peripheral glucose uptake and glycogen synthesis. Xylitol infusion during insulin clamp studies in normal rats prevented fatty-acid-induced insulin resistance, with favourable effects on glycogen synthesis and insulin-mediated glucose uptake.

The potential favourable effects of exogenous xylitol administration on glucose homeostasis may be explained by known aspects of xylitol metabolism. For example, the glycaemic index of xylitol is much lower than that of glucose, a property that makes it more favourable if the caloric load needs to be better controlled, as in PD patients. Importantly, in human xylitol is a very poor insulin-secretagogue compared to glucose [95]. Indeed, it is well known that hyperinsulinemia per se is probably one of the most potent drivers of insulin resistance [99, 100] and that a constant peritoneal daily glucose load may facilitate hyperinsulinemia in PD patients. On the other hand, it has been shown both in animal models and humans that insulin resistance and diabetes may be efficiently counteracted by K-ATP channel openers, like diazoxide, which inhibits β-cell insulin secretion [99].

As mentioned above, a key intermediate of liver xylitol metabolism is D-xylulose-5-phosphate (Xy-5-P), a compound that affects the concentration of fructose 2,6-bisphosphate (F 2,6-P2), the most potent activator of phosphofructokinase and hence glycolysis, especially in liver [101]. Synthesis and degradation of F2,6-P2 are catalysed by a bifunctional enzyme, Fru-6-P,2-kinase: Fru-2,6-bisphosphatase, which in turn is regulated by a cAMP-dependent protein kinase and a protein phosphatase 2A. When the kinase is activated, gluconeogenesis is stimulated via a decrease of F 2,6-P2, whereas the phosphatase is activated to stimulate glycolysis via an increase of F 2,6-P. Since Xy-5-P is an allosteric activator of protein phosphatase 2A, this results in a rapid increase in F 2,6-P2, activation of phosphofructokinase, thus activating glycolysis, and inhibiting gluconeogenesis [102]. This is consistent with the observation that the infusion of xylitol during a combination of a pancreatic and hyperglycemic clamp leads to a significant elevation of Xy-5-P. Interestingly, xylitol also induces a marked decrease of phosphonelpyruvic carboxykinase (PEPCK) mRNA in the liver, suggesting that Xy-5-P may also depress gluconeogenesis via a direct transcriptional effect [103].

### Use of Xylitol in PD solution

*Biocompatibility profile* The biocompatibility and toxicity of a PD solution containing d-xylitol in different concentrations compared to glucose was examined in mesothelial cells in vitro [Arduini A, Patent PCT/EP2006/060162]. Main findings were: an increase in the number of mesothelial cells in the presence of solutions with low or absent glucose; higher concentrations of phospholipids and phosphatidylcholine (indispensable surfactants for normal functioning peritoneum) in the presence of d-xylitol; lower formation of giant cells and no increase in interleukin 1 (expression of cellular injury) with PD fluid containing xylitol.

*Osmotic capacity.* Although data for d-xylitol are scanty compared to those for l-carnitine, fluid transport across peritoneum comparable to l-carnitine is likely given the closeness of their molecular weight. In a clinical trial many years ago [104], six insulin-dependent diabetic patients on CAPD were switched from a glucose-based PD regimen to a daily therapeutic program consisting of the use of d-xylitol as the sole osmotic agent (three daily exchanges of PD solution with xylitol 1.5% and one exchange with xylitol 3%). After a follow-up of five months, fluid balance (as indicated by peritoneal ultrafiltration, body weight, and mean arterial pressure) obtained with xylitol-containing PD solution was comparable to that obtained with glucose use [104]. *Metabolic effects* In the clinical trial conducted by Bazzato et al. in ESRD insulin-dependent diabetics on CAPD [104], the therapeutic schedule, followed for a median duration of 8.7 months (range 5–11 months), consisted in 4 daily exchanges of 2L PD solution, from which 3 were with normal osmolality solution (xylitol 1.5 g/dL) and one with hyperosmolar solution (3 g/dL). The total daily dose of xylitol administered via peritoneum with this protocol was 150 g. The plasma xylitol level was 30 mg/dL during the daily dwells and reached 80 mg/dL during the nocturnal dwell. These concentrations had no influence on plasma osmolality. The required exogenous insulin was reduced by 50% compared to the amount administered during CAPD with glucose. HbA1c was significantly lower during treatment with xylitol as compared to the therapeutic period with glucose, indicating better glycemic control in diabetic patients [104]. It should be noted that the extent of the improvement of glycemic control in the CAPD patients treated with xylitol was remarkable (from 12.99 to 10.72% HbA1C) and cannot be solely explained by glucose-sparing action. Indeed, as discussed above, xylitol metabolic by-product like Xy-5-P is a potent allosteric and transcriptional activator favourably affecting liver glucose homeostasis in insulin resistance and diabetes (see above).

In treated patients there was a significant improvement in the lipid profile: triglycerides and total cholesterol levels decreased after the first two months of treatment, while HDL-cholesterol increased, restoring normal levels after 2–5 months. All these parameters remained stable in the month subsequent to the study. The plasma concentration of inorganic phosphorus, compromised during CAPD with glucose, was restored in months of treatment. During the observation period, a progressive increase of serum uric acid concentration was found in PD patients treated with PD solution containing xylitol, requiring administration of allopurinol that induced a slow reduction of these values [104]. Lactic acid was also enhanced during the entire treatment period, remaining however within normal range. The dosage of 150 g/day xylitol was well tolerated, without evidences of adverse effects or clinical signs of calcium oxalate deposition. Only when the xylitol dosage exceeded 150 g/day, in patients who needed PD solutions with higher content of xylitol in order to augment the ultrafiltration effect, adverse events like nausea, vomiting or increased levels of serum bilirubin and transaminases occurred [104].

The use of xylitol as partial substitute of glucose in peritoneal dialysis bags was more recently examined by employing a 2.5% (w/v) xylitol-based PD solution for the long-dwell (nocturnal) exchange, with 50 g of instilled xylitol (Buoncristiani U, personal communication, 2004). The beneficial effects of xylitol-based PD solution were a lower 24-h glycemic curve and a reduced response of insulin. Moreover, the lipid metabolism seemed to be improved and an increase of serum protein and albumin was found. Two patients have used such xylitol-based PD solution for 10 years and only a slight increase in serum uric acid levels, which however remained within normal range, was observed (Buoncristiani U, personal communication, 2004).

## Combining l-carnitine and Xylitol in PD fluid

The concept of introducing more than one osmo-metabolic agent to dialysate is derived from the approach of polypharmacy or combination therapy, whereby the aim is to achieve a favorable synergetic action. We have recently examined the biocompatibility of a new solution containing l-carnitine (1.24 mmol/L), xylitol (46 or 98.8 mmol/L), and low-dose glucose (27.7 mmol/L) [66]. Maximum xylitol dosage patients would be exposed to is 36 g/day, similar or lesser than that used by Buoncristiani (50 g/day), and much less than that used by Bazzato (150 g/day) [104].

The new solution was compared to low-GDPs glucose-based PD solution and tested in cultured HUVECs obtained from the umbilical cords of healthy and gestational diabetic mothers. Gestational diabetes is associated with inflammation, oxidative stress, and overexpression of inflammatory cytokines, which represent common abnormalities in patients on PD. In both endothelial cell types, the use of experimental PD solution did not change the proliferative potential and significantly improved cell viability. Glucose-based solutions significantly increased the membrane expression of Intracellular Adhesion Molecule-1 (ICAM-1) and Vascular Adhesion Molecule-1 (VCAM-1). Such pro-inflammatory vascular effect may promote adhesion of monocytes to endothelial cells, a crucial event in the early atherosclerotic process [105]. Indeed, a significant increase in monocyte interaction with HUVECs was found with glucose-based PD fluids [66]. All such unfavorable vascular effects were absent when endothelial cells were exposed to the experimental PD solution. To be noted that, although the test solution contained some glucose, it was at a lower concentration and did not seem to have the deleterious effects the higher concentration did. Thus, a small amount of glucose may be maintained in the formulation of the PD solution, in order to take advantage of its ultrafiltration ability and energy-providing potential.

Two clinical trials with new PDSs based on l-carnitine, xylitol, and low glucose are under advanced development. FIRST (efficacy and saFety assessments of a peritoneal dIalysis solution containing glucose, xylitol and l-CarRnitine compared to standard PD SoluTions in CAPD) is an ongoing 1-month study designed to evaluate safety, tolerability, and efficacy of the proposed new PD solution (NCT04001036). The ELIXIR trial (a study to EvaLuate the effIcacy and safety of Xylocore, a glucose sparing expeRimental solution for PD) is a randomized, controlled, parallel groups, international multicenter study, whose primary objective is the non-inferiority of the experimental solution compared to a glucose-based low-GDP PD solution with regard to safety and efficacy, over a 6-month treatment period (NCT03994471).

## Conclusions

The limitations of the currently available glucose-based solutions for PD are widely recognized. Exposure to significant amounts of glucose during the course of PD is of concern for both local and systemic detrimental effects [22], and it is thought that glucose sparing should mitigate these effects [4].

An ideal biocompatible solution for PD should deliver prolonged and sustained ultrafiltration and solute clearance, not influence the normal peritoneal morphology and physiology, not induce systemic adverse effects related to absorption, and, last but not least important, provide beneficial metabolic and nutritional effects, if absorbed.

The two main forms of PD have some peculiar differences: CAPD (manual) composes of 4–5 exchanges with long dwell times around the day, whereas APD (automated) is usually performed during the night (8–10 h duration) with short dwell time cycles, which can be “extended” with one or two long dwell during the rest of the day (14–16 h duration). It should also be taken into account that PD solutions using osmo-metabolic agents, capable to reduce glucose exposure and/or attenuate its systemic effect, may have different impact, depending on the modality (CAPD or APD) and the prescription, to sustain the osmotic gradient over the dwell time the peritoneal cavity is exposed to the PD solution. Likewise, any PD treatment aimed to reduce, among others, glucose exposure may further benefit in using osmo-metabolic agents. This is the case of incremental PD, a dialysis strategy that is in keeping with the issue of green nephrology [106], and may offer several advantages with no evidence of any harmful effect [107, 108].

**Fig. 3:**
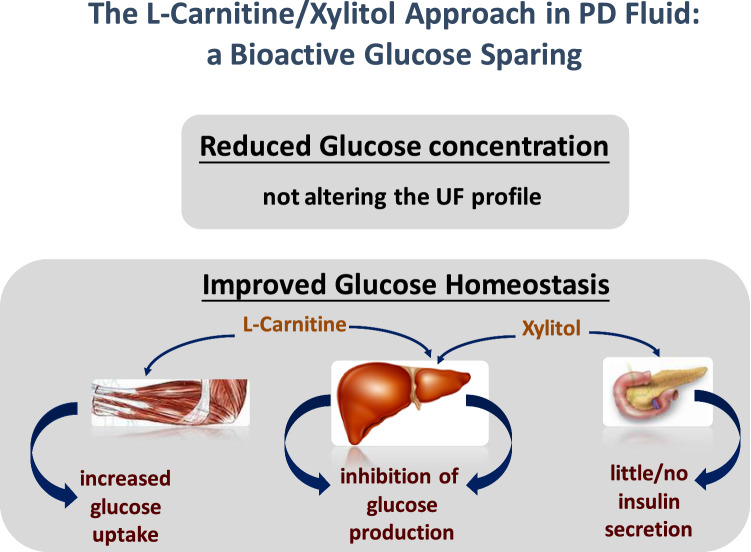
Potential benefits of the osmo-metabolic approach in PD fluid

Data reviewed in this article suggest that a PD solution containing osmo-metabolic agent, besides lower concentrations of glucose when compared to the glucose-based present PD solutions, may not only significantly decrease the amount of glucose currently present in commercial PD solutions, but also take advantage of the described metabolic properties, and correct potential metabolic deficiencies or abnormalities.

l-carnitine and d-xylitol are two concrete examples of such agents (Fig. 3); a synergistic effect by the two compounds in terms of improving glucose and lipid homeostasis in PD patients can be anticipated, and this in turn may significantly attenuate cardiovascular morbidity in both diabetic and non-diabetic patients on PD therapy. The d-xylitol-d-glucose-l-Carnitine-based PD solution is also characterized by a more biocompatible profile than d-glucose-based ones [66].

It is essential to address another important outcome aspect: the cost-benefits of the PD solutions in the market. While the “established glucose-sparing PD solutions” (icodextrin, amino acids) are widely used in the developed world with both clinical as health economic proven benefits to the patients and the society, most of the countries in development have either a very limited or no access at all to these glucose-sparing PD solutions, due to their cost. Another benefit of the osmo-metabolic approach could be that these “newcomer” PD solutions, besides showing their competitive potential in regard to efficiency, effectivity, clinical and safety aspects, they might also broaden the offer in relation to the demand.

Osmo-metabolic agents can be used alone, or in combination, to maximize their therapeutic effects. The results of the FIRST and ELIXIR studies will clarify whether the osmo-metabolic agents approach promising results are borne out in large randomized studies. Future research should also be directed to the identification of additional osmo-metabolic agents and how to combine them to better address not only preservation of peritoneal membrane and residual kidney functions, but also underlying comorbidities able to increase cardiovascular risk.
